# Risk Factors and Clinical Characteristics of Pandrug-Resistant Pseudomonas aeruginosa

**DOI:** 10.7759/cureus.58114

**Published:** 2024-04-12

**Authors:** Shahed Kamal, Karan Varshney, Danielle J Uayan, Bettina G Tenorio, Preshon Pillay, Sergiu T Sava

**Affiliations:** 1 Internal Medicine, Northern Hospital Epping, Melbourne, AUS; 2 Public Health, School of Medicine, Deakin University, Waurn Ponds, AUS; 3 Medicine, Ateneo School of Medicine and Public Health, Manila, PHL; 4 Medicine, Ateneo School of Medicine and Public Health, Philippines, Manila, PHL; 5 Faculty of Medicine and Dentistry, University of Alberta, Alberta, CAN; 6 Medicine, School of Medicine, Deakin University, Geelong, AUS

**Keywords:** pseudomonas, antibiotic resistance, scoping review, pseudomonas aeruginosa, pandrug-resistance

## Abstract

The emergence of increasingly resistant strains of *Pseudomonas aeruginosa *is a great public health concern. Understanding the risk factors and clinical characteristics of patients with pandrug-resistant *P. aeruginosa *(PDR-PA) can help inform clinicians in creating guidelines for both prevention and management.

Using Preferred Reporting Items for Systematic Reviews and Meta-Analyses (PRISMA) guidelines, this scoping review retrieved existing literature on PDR-PA by searching PubMed, SCOPUS, Embase, Web of Science, and CINAHL databases. From the 21 studies that satisfied the inclusion criteria,1,059 *P. aeruginosa* samples were identified, and 161, or 15.2% of the isolates were found to have pandrug resistance. Furthermore, our review suggests that PDR-PA was largely hospital-acquired, and patients suffering from burn injuries and chronic lung diseases had a higher risk of colonization than other hospitalized individuals. In five out of the 21 studies, administration of the antibiotic colistin emerged to be the preferred therapeutic strategy. With regards to concurrent infections, *Acinetobacter *and *Klebsiella *species were found to occur most frequently with PDR-PA, suggesting mutualistic interactions that enable further antimicrobial resistance.

In conclusion, this review showed the prevalence of PDR-PA and outlined the demographic and clinical profile of affected patients. Further research is needed to investigate the transmission and outcomes of PDR-PA infections and to find potential therapeutic strategies.

## Introduction and background

*Pseudomonas aeruginosa* is a monoflagellated, non-spore-forming Gram-negative facultative aerobe that is found ubiquitously, especially in freshwater sources [1]. It can survive broad temperature spectrums, distinguishing it from other Pseudomonas species [2]. The 2019 report from the Global Burden Disease Study, which collected surveillance data from 343 million individual records worldwide, showed that *P. aeruginosa* was the second most common pathogen responsible for both community-acquired pneumonia and hospital-acquired pneumonia [3]. It was also found to be the fifth highest cause of infection-related deaths out of all investigated bacterial pathogens [3]. 

Severe infections of *P. aeruginosa* often arise in patients with burn injuries, immunocompromised individuals, or those who have chronic conditions such as cystic fibrosis (CF), chronic obstructive pulmonary disease (COPD), and non-healing wounds from diabetes [4]. In fact, in adults afflicted with CF, *P. aeruginosa* was the most prevalent pathogen isolated in cultures, contributing to a 2.6-fold increase in the eight-year risk of death [5,6]. Due to *P. aeruginosa*’s capability of forming biofilms with other microbes, patients in ventilators, catheters, and other implantable devices were also found to be at higher risk of mortality than other hospitalized individuals [4].

First-line treatment often includes antipseudomonal penicillin, carbapenems, fluoroquinolones, cephalosporins with antipseudomonal activity, and monobactams, as guided by in vitro testing on isolates to determine susceptibility [7,8]. Unfortunately, *P. aeruginosa* has demonstrated marked resistance to many antibiotics and has been included in the “critical” category in the World Health Organization’s global list of priority pathogens for investigation [9]. *P. aeruginosa* has been classified according to the degree of resistance: multidrug-resistant (MDR), extensively drug-resistant (XDR), and pandrug-resistant (PDR). A consensus for the designations of MDR, XDR, and PDR microorganisms was released in 2012, and they are defined as follows: MDR microbes should be non-susceptible to ≥1 agent in >3 antimicrobial categories; XDR is defined as non-susceptible to ≥1 agent in all but ≤2 categories; and PDR is defined as non-susceptible to all antimicrobial agents [10]. 

Patterns of resistance exist throughout various regions of the world; a study of isolates from countries in the Asia-Pacific region between 2015 and 2019 reported rates of 20.5% of MDR infections [11]. Within Europe, 33.9% of *P. aeruginosa* infections were resistant to at least one antimicrobial class [7]. Past infection and antibiotic exposure, often seen in the ICU setting, is a risk factor for acquiring MDR or XDR *P. aeruginosa* infection [12-14]. Other risk factors include length of ICU stay, prolonged bedridden status, surgery, certain implantable devices, and if the previous room occupant was colonized by the same infectious pathogen [15,16]. Given the organism’s high levels of resistance, along with its high variability of the resistance patterns among regions, targeted antimicrobial therapy is heavily preferred for management [7,8]. 

In more serious infections or suspected MDR infections, combination therapies are used empirically while awaiting susceptibility testing results, since a delay in treatment is associated with increased mortality [7,8]. If there is resistance to first-line agents, combination drugs such as ceftolozane-tazobactam and ceftazidime-avibactam, which have shown benefit in some studies, may be used [7]. If there is further resistance to these medications, other alternatives, which are still yet to be extensively studied, include cefiderocol and imipenem-cilastatin-relebactam [7]. Newer β-lactams/β-lactamase inhibitor combinations are also being developed as treatment options, as well as a novel antibiotic, murepavadin, which has showcased encouraging clinical outcomes [8].

Drug-resistant *P. aeruginosa*, particularly PDR *P. aeruginosa* (PDR-PA), poses a considerable threat to public health due to its non-susceptibility to all existing antibacterial substances. However, the epidemiology and risk factors for PDR-PA are still poorly understood; to further complicate the problem, there is still debate around the proper use of the term “pandrug-resistant” [17-19]. Therefore, we aim to conduct a scoping review of the literature on PDR-PA with two primary objectives: to determine the clinical and epidemiological factors in cohorts where PDR-PA has emerged, and to provide insights into how PDR-PA is defined in the literature. By doing so, this review can offer important insights into this major public health problem and can potentially guide treatment, especially for those who are at the highest risk. 

## Review

Methodology

For this review, the Preferred Reporting Items for Systematic Reviews and Meta-Analyses extension for Scoping Reviews (PRISMA-ScR) [20] were followed. Searches were conducted across five different databases on March 7, 2023: PubMed, SCOPUS, Embase, Web of Science, and Cumulative Index of Nursing and Allied Health Literature (CINAHL). Search terms focused on *P. aeruginosa* and pandrug resistance. Search terms used in databases were (“pan-resistant” OR “pan resistant” OR “pan drug-resistant” OR “pan-drug resistant”) AND (“pseudomonas aeruginosa” OR “*P. aeruginosa*” OR “pseudomonas”).

After the initial searches were conducted, duplicates were removed. Thereafter, articles were screened by title and abstract. Next, articles were further screened by full-text analysis. As per the PRISMA-ScR guidelines, articles were independently screened by multiple reviewers. Any discrepancies in articles considered eligible for inclusion were resolved by consensus among reviewers.

To capture as many relevant studies as possible, no restrictions were placed based on the date of publication. Our inclusion criteria were as follows: (1) must include at least one patient with PDR-PA, (2) must describe clinical characteristics/outcomes or at least one modifiable risk factor for patients, (3) must be in English, (4) must be original research, and (5) must have stratified data for PDR-PA patients. Studies were excluded if they were non-clinical studies, were in a language other than English, and were dissertations, conference abstracts, commentaries, editorials, or reviews.

For the articles that were deemed to be eligible for inclusion, data were extracted by multiple reviewers independently; again, any contention amongst reviewers was resolved by consensus. The main data extracted were the study characteristics, patient characteristics, and outcomes. For study characteristics, the following data were extracted: year of publication and data collection period, study design, location, sampling method, target population, and total study sample size. Age, sex, infection onset, site of isolation of causative microbe, comorbidities, and the presence of concurrent infection were also determined. If available, the total amount of deaths amongst PDR-PA patients, interventions, and definitions for PDR-PA were noted. Extracted data were presented in tabular format; trends in data and pertinent findings were described in the text, with the authors utilizing a narrative synthesis.

Results

Study Selection

**Figure 1:**
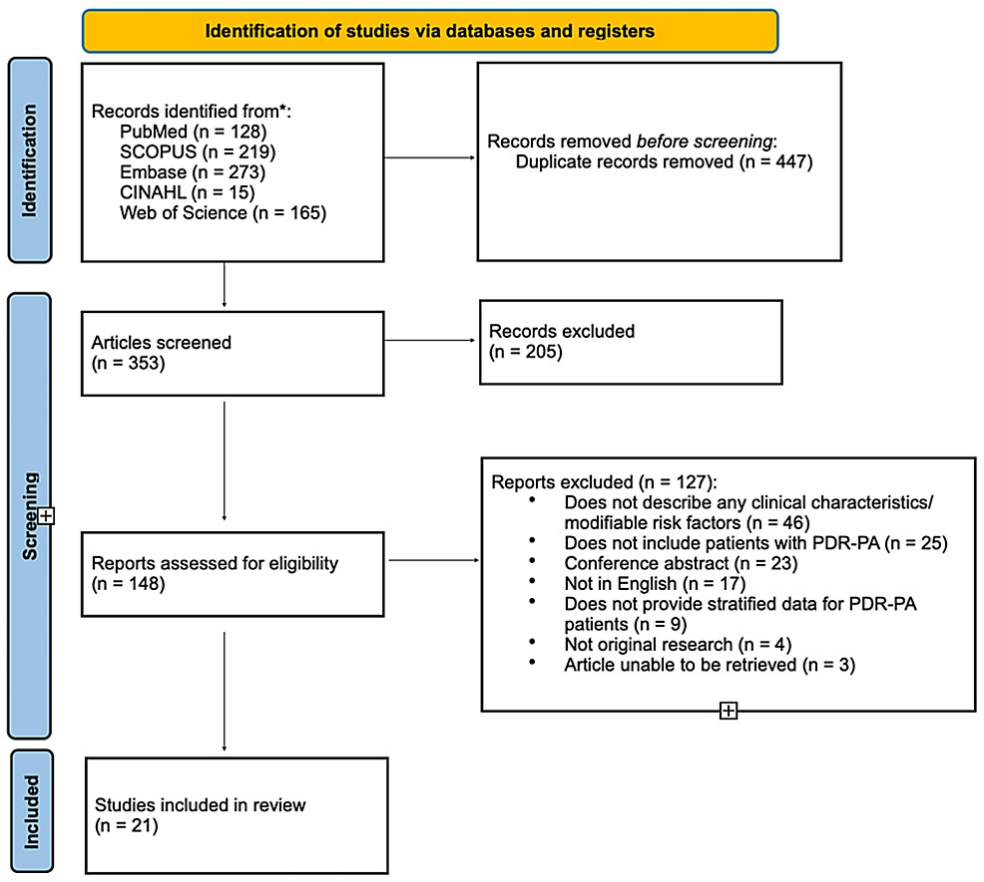
Study selection process based on the PRISMA 2020 flow diagram

Searches from the databases produced a total of 800 articles. A total of 447 duplicates were removed, and 353 articles were then reviewed by title and abstract. At that stage, the most common reason for the exclusion of articles was due to studies not having patients with PDR-PA. The remaining 148 articles were screened by reading the full text. Ultimately, 21 articles were deemed to be eligible for inclusion [21-41]; the most common reasons for exclusion in this review were the absence of data on PDR-PA specifically, and the lack of information on patients’ clinical characteristics or risk factors. A full depiction of the workflow for the screening process is shown in Figure 1 [20].

Publication Types, Geography, and Target Population

Table 1 describes the characteristics of the articles included in this review. A substantial number of studies that investigated the emergence of PDR-PA were published in India (n = 5), followed by the United States (n = 3), Iran and Greece (n = 2). The publication dates also ranged widely, the earliest dating from 2004 and the latest published in 2020. A significant proportion of included studies had an observational design and utilized convenience sampling to recruit patients. Moreover, eight studies had a cross-sectional design, five were cohort studies, six were case reports or series, and three were chart reviews. Sample size also varied depending on the study type conducted: predictably, case reports only described PDR bugs in one patient, while cross-sectional and cohort studies investigated an aggregate of 995 isolates and 2,230 patients. 

**Table 1:** Study characteristics

Author (year)	Country	Location	Data Collection Period	Study Type	Sampling Method	Diagnostic Method Used	Target Population	Total Sample Size
Kofteridis (2020)	Greece	Crete	2010 - 2015	Retrospective cohort	Convenience	Disc diffusion, Vitek 2 system	Inpatients with PDR infections	158 isolates
Gupta (2019)	India	Jharkhand	2015 - 2017	Cross-sectional	Convenience	Vitek 2 system	Burns	513 isolates
Mohamed (2019)	Sudan	Khartoum	Unspecified	Case study	Purposive	Disc diffusion, Whole genome sequencing	Postoperative infection	384 patients
Winstead (2019)	USA	Kentucky & California	2008 - 2016	Retrospective cohort	Convenience	Unspecified	Cystic Fibrosis patients who underwent lung transplantation	118 patients
Aljanaby (2018)	Iraq	Al-Najaf	2015 - 2017	Cross-sectional	Convenience	Disc diffusion	Burns	30 patients
Arumugam (2018)	India	South India	2012 - 2015	Cross-sectional	Convenience	Disc diffusion, spectrophotometer	Unspecified	44 patients
Castañeda-Montes (2018)	Mexico	Mexico City	1996 - 2007	Cross-sectional	Convenience	Vitek 2 system	Cystic Fibrosis, Neonatal Sepsis, Nosocomial Infections	36 patients
Gashaw (2018)	Ethiopia	Jimma	2016	Cross-sectional	Convenience	Disc diffusion	Healthcare-associated infections	13 patients
Molinaro (2018)	Italy	Unspecified	Unspecified	Case Study	Purposive	Unspecified	Meningitis	144 isolates
Alipour (2017)	Turkey	Ankara	2013	Case-series	Purposive	Disc diffusion, E-test	Pulmonary infection from contaminated bronchoscope	1 patient
Khosravi (2016)	Iran	Ahvaz	2012	Cross-sectional	Convenience	Disc diffusion, PCR amplification of target resistance genes, microdilution	Burns and local wound	54 patients
Dimopoulos (2015)	Greece	Multiple	2008 - 2009	Prospective cohort	Convenience	Antimicrobial Susceptibility Testing	ICU patients	150 isolates
Fernandes (2015)	India	Visakhapatnam	2009 - 2013	Retrospective chart review	Convenience	Disc diffusion	Bacterial keratitis	30 isolates
Kulkova (2015)	Slovakia	Bratislava	2011 - 2012	Cross-sectional, Case report	Purposive	Disc diffusion	Unspecified	15 patients
Yang (2015)	China	Shanghai	2011 - 2013	Case-series	Convenience	Disc diffusion	Unspecified	1 patient
Gibson (2014)	Ireland	Dublin	2012	Case report	Purposive	Unspecified	Cystic fibrosis	1 patient
Friedstat (2013)	USA	Massachusetts	2007 - 2010	Retrospective chart review	Convenience	Unspecified	Pediatric Burns	14 patients
Movahedi (2013)	Iran	Tehran	2010	Cross-sectional	Simple Random	PCR amplification of target resistance genes (ERIC-PCR)	Cystic Fibrosis	299 patients
Babu (2011)	India	Davangere	Unspecified	Prospective cohort	Convenience	Disc diffusion	Intubated patients	288 patients
Goverman (2007)	USA	Boston	1990 - 2005	Retrospective chart review	Convenience	Disc diffusion, Microdilution	Pediatric Burns	65 patients
Dobbin (2004)	Australia	Sydney	1989 - 2002	Retrospective cohort	Convenience	Disc diffusion, Microdilution	Cystic Fibrosis patients who underwent lung transplantation	870 patients

Many studies investigated PDR organisms in burn and CF patients, having five studies each. Two studies focused on CF patients who underwent lung transplant patients [24,41], and both found *P. aeruginosa* as the most frequent microbe isolated that had PDR. All five burn studies also found similar results [22,25,31,37,40]. Notably, one pediatric burns study demonstrated a significantly higher prevalence of PDR-PA in international patients compared to their USA counterparts [37]. 

Patient Demographics and Infection Characteristics

The patient demographics and clinical characteristics are listed in Table 2. Patient profiles tended to vary. For example, the ages ranged widely from 0 to 90 years old. PDR organisms were isolated more often in males (55%, n = 762) than in females (42.8%, n = 615). Patients presented with varying comorbidities, namely CF and other respiratory problems (42%, n = 9), diabetes mellitus (14.3%, n = 3), cardiovascular disease (14.3%, n = 3), and immunosuppression (9.5%, n = 2). As a significant number of studies from our review pool focused on inpatient, CF and burn patients, PDR isolates were frequently taken from burn wounds, sputum, and blood samples. PDR-PA infections were often observed to have started at the hospital.

**Table 2:** Patient demographics and clinical characteristics

Author (year)	Target Population	Age	Sex	Infection Onset	Site of Isolation	Comorbidities	Concurrent Infections
Kofteridis (2020)	Inpatients with PDR infections	45.5 - 74.5	Male: 49 Female: 16	Hospital-onset	Sputum, blood, urine, pus, ascites, cerebrospinal fluid	Chronic heart disease, chronic lung disease, malignancy, diabetes mellitus, chronic renal disease	Acinetobacter sp., Klebsiella sp.
Gupta (2019)	Burns	1 - 79	Male: 130 Female: 169	Hospital-onset	Burn wound	Unspecified	K. pneumoniae, A. baumannii, E. coli, Enterobacter sp., Pseudomonas luteola, B. cepacia, S. aureus, P. mirabilis, E. cloacae
Mohamed (2019)	Postoperative infection	52	Female: 1	Hospital-onset	Postoperative wound	Unspecified	Unspecified
Winstead (2019)	Cystic Fibrosis patients who underwent lung transplantation	15 - 44	Male: 22 Female: 22	Mixed	Unspecified	Cystic fibrosis	MRSA, MSSA, S. maltophilia, Achromobacter xylosoxidans, Enterococcus sp., B. gladioli, M. abscessus, S. marcescens
Aljanaby (2018)	Burns	18 - 45	Unspecified	Hospital-onset	Burn wounds	Unspecified	MRSA, MSSA, K. pneumoniae, E. coli, A. baumannii, S. typhi
Arumugam (2018)	Unspecified	Unspecified	Unspecified	Unspecified	Unspecified	Unspecified	Not tested
Castañeda-Montes (2018)	Cystic Fibrosis, Neonatal Sepsis, Nosocomial infections	Unspecified	Unspecified	Mixed	Blood, sputum, stool, catheter	Cystic fibrosis	Unspecified
Gashaw (2018)	Healthcare-associated infections	Unspecified	Unspecified	Hospital-onset	Blood, urine, wound, pus, sputum	Cardiovascular disease, hypertension, diabetes mellitus, HIV, severe malnutrition	S. aureus, E. coli, Klebsiella sp., Citrobacter, Enterobacter, Proteus sp., Providencia sp., A. baumannii, Serratia sp.
Molinaro (2018)	Meningitis	66	Female: 1	Hospital-onset	Cerebrospinal fluid	Cavernous angioma	Not tested
Alipour (2017)	Pulmonary infection from contaminated bronchoscope	38 - 69	Male: 10 Female: 5	Hospital-onset	Bronchi	Unspecified	Unspecified
Khosravi (2016)	Burns and local wound	18 - 73	Male: 75 Female: 75	Mixed	Burn wounds	Unspecified	Not tested
Dimopoulos (2015)	ICU patients	59.5 (mean)	Male: 186 Female: 102	Mixed	Blood	Respiratory infection, sepsis, Cardiovascular diseases, Immunosuppression, Hepatic Diseases	Acinetobacter sp., Klebsiella sp., Pseudomonas sp., Enterococcus sp., Candida albicans, Candida non-albicans
Fernandes (2015)	Bacterial keratitis	Unspecified	Unspecified	Community-onset	Eye discharge	Glaucoma	Not tested
Kulkova (2015)	Unspecified	Unspecified	Unspecified	Mixed	Unspecified	Pneumonia	A. baumannii, E. faecalis
Yang (2015)	Unspecified	43 - 90	Male: 21 Female: 9	Mixed	Sputum, urine, pus, blood	Pulmonary infections, urinary tract infection, local wound infection, blood infection, gallbladder infection	A. baumannii, K. pneumoniae
Gibson (2014)	Cystic fibrosis	31	Male: 1	Mixed	Sputum	Cystic Fibrosis, Crohn's Disease	Unspecified
Friedstat (2013)	Pediatric Burns	8.23 (mean for international patients); 5.72 (mean for US patients)	Male: 209; Female: 175	Mixed	Burn wounds, blood	Urinary Tract Infection	A. baumannii, E. cloacae, S. marcescens, K. pneumoniae, E. coli, S. maltophilia, C. meningosepticum, B. cepacia, C. meningo, Citrobacter sp., F. odoratum, K. oxytoca, A. lwoffii
Movahedi (2013)	Cystic Fibrosis	2.79 (mean)	Male: 19; Female: 17	Mixed	Endotracheal aspirates, wound, urine, blood, sputum	Cystic fibrosis	Not tested
Babu (2011)	Intubated patients	Unspecified	Unspecified	Hospital-onset	Endotracheal aspirates	Diabetes mellitus, malignancy, chronic lung disease	K. pneumoniae, A. baumannii, E. coli, S. aureus
Goverman (2007)	Pediatric Burns	5 - 17	Male: 11 Female: 3	Mixed	Blood, lung, urine, burn wounds	Unspecified	A. baumannii, S. aureus, K. pneumoniae, S. marcescens, S. maltophilia, E. cloacae, P. stuartii, C. meningosepticum, P. mirabilis, E. aerogenes, C. freundii
Dobbin (2004)	Cystic Fibrosis patients who underwent lung transplantation	29 (mean)	Male: 29 Female: 25	Hospital-onset	Sputum, bronchial washings, tissue, blood	Cystic fibrosis	B. cepacia, MRSA

Co-infections with organisms other than *P. aeruginosa* were also found in several studies. In terms of frequency, *Acinetobacter baumannii* and other Acinetobacter species were discovered to be the most prevalent (n = 11), followed by *Klebsiella *(n = 10) and Staphylococcus species (n = 9). Other concurrent infections found among patients were Enterobacter (most commonly *E. clocae*), Burkholderia (*B. cepacia*), Serratia (*S. marcsecens*), and Proteus (*P. mirabilis*). 

Prevalence, Outcomes, and Definitions of PDR-PA

Through reviewing how “pandrug-resistant,” “multidrug-resistant,” and “extensively drug-resistant” bugs were classified from each other across all studies, three main concepts emerged as the basis for PDR definitions (Table 3). PDR organisms were most commonly described as resistant to all commercially available antimicrobial classes (57.1%), resistant to all antimicrobials tested in the study (23.8%), or resistant (or sensitive only) to colistin (14.2%).

**Table 3:** Differences in PDR definitions, PDR-PA frequency, interventions, and mortality

Author (year)	Definition of PDR	Total PRD-PA / PA samples (%)	Interventions	Deaths with PDR-PA / Total Cases of PDR-PA (%)
Kofteridis (2020)	"Non-susceptibility to all agents in all antimicrobial categories"	6 / 6 (100)	IV Colistin + Tigecycline + Carbapenems	Unspecified / 6
Gupta (2019)	"Non-susceptibility to all agents in all antimicrobial categories"	11 / 67 (16.4)	Unspecified	Unspecified / 11
Mohamed (2019)	"Showed resistance to 18 of antibiotics tested"	1 / 1 (100)	Unspecified	Unspecified / 1
Winstead (2019)	"Resistant to all antimicrobials"	2 / 38 (5.3)	Antibiotics*	Unspecified / 2
Aljanaby (2018)	"Resistance to all antimicrobial class"	8 / 142 (5.6)	Unspecified	Unspecified / 8
Arumugam (2018)	Unspecified	7 / 144 (4.9)	Unspecified	Unspecified / 7
Castañeda-Montes (2018)	"Non-susceptible to any antimicrobial agent"	15 / 158 (9.5)	Unspecified	Unspecified / 15
Gashaw (2018)	"Resistant to all antimicrobials"	3 / 9 (33.3)	Unspecified	Unspecified / 3
Molinaro (2018)	"Resistant to all cephalosporins, piperacillin–tazobactam, aztreonam, carbapenems, ciprofloxacin, and aminoglycosides"	1 / 1 (100)	IV Amikacin	0 / 1 (0)
Alipour (2017)	"Resistance to colistin"	15 / 15 (100)	Unspecified	Unspecified / 15
Khosravi (2016)	"Resistance against all types of antibiotics	1 / 150 (0.6)	Unspecified	Unspecified / 1
Dimopoulos (2015)	"Resistant to all available antimicrobials"	1 / 40 (2.5)	IV Meropenem, IV Tigecycline, IV Colistin	Unspecified / 1
Fernandes (2015)	"Resistant to all groups of antibiotics"	1 / 13 (7.7)	Imipenem, Azithromycin, Imipenem + Colistin	1 / 13 (7.6)
Kulkova (2015)	"No susceptibility to any of the antibiotics tested"	1 / 16 (6.2)	IV Meropenem + Vancomycin + Fluconazole + Metronidazole	0 / 1 (0)
Yang (2015)	"Resistant to all antimicrobials"	5 / 5 (100)	Antibiotic regimens**	Unspecified / 5
Gibson (2014)	"Sensitive only to tobramycin and colistin"	1 /1 (100)	Immunosuppression (IV Infliximab)	0 / 1 (0)
Friedstat (2013)	"Bacteria resistant to all tested antibiotics"	12 / 42 (28.6)	IV Colistin	9 / 12 (75)
Movahedi (2013)	"Resistant to all antimicrobials"	13 / 49 (26.5)	Unspecified	6 / 13 (46.1)
Babu (2011)	"Resistant to all antibiotics"	16 / 96 (16.7)	Unspecified	Unspecified / 16
Goverman (2007)	"Resistance to all tested antibiotics"	13 / 14 (92.8)	IV Colistin	2 / 14 (14.3)
Dobbin (2004)	"Any isolate that was resistant to each tested antibiotic"	28 / 52 (53.8)	Immunosuppression therapy***	6 / 28 (21.4)

From the total of 1,059 *P. aeruginosa* samples, 161 were found to have pandrug-resistance (15.2%). Affected patients were oftentimes given intravenous antibiotics, namely colistin, and several carbapenems. Mortality from PDR-PA cannot be determined as most studies did not attribute deaths specifically to it. 

Discussion

Despite the growing efforts for antimicrobial stewardship and evidence-based practices to prevent nosocomial infections, there appears to be an increasing dissemination of PDR-PA especially in vulnerable populations. Most of the journals showed hospitalized and immunocompromised patients to be particularly at risk, but PDR-PA was also found to have outbreak potential as evidenced by one of the studies reviewed; a single contaminated bronchoscope was able to infect 14 other patients that were otherwise asymptomatic before the procedure [30].

Therefore, to prevent its spread, we must first expand our current knowledge on resistant organisms, as well as settle on a universal definition for pandrug-resistance. From our analysis, nearly all studies employed the literal definition of the prefix “pan,” which means “all” in Greek [42], with most directly citing the consensus definition of PDR from Magiorakos et al. (2012). However, three studies classified PDR organisms based on their resistance or sensitivity to colistin [29,30,36]; it is also noteworthy that these articles were published beyond the year the consensus was released [10]. To avoid further confusion and the subsequent inappropriate use of antibiotics, it is important that terminologies be agreed upon and applied consistently throughout the literature. We recommend a departure from the definition of PDR as “resistance or susceptibility to polymyxins (i.e., colistin)” since the use of the prefix “pan” implies resistance to all available antimicrobial agents.

This scoping review revealed a worrying prevalence of PDR-PA amongst *P. aeruginosa* infections at 15.2%. Our review suggests that PDR-PA was overwhelmingly hospital-acquired, and although antimicrobial resistance is a problem worldwide, certain regions carried a higher burden for PDR organisms. Moreover, burn patients and those with chronic diseases are particularly at risk, with their immunocompromised states increasing their overall susceptibility to nosocomial pathogens. Addressing this spread requires a greater understanding of resistance transmission and providing systemic changes to eradicate most if not all root causes. For lower-middle-income countries like India and Iran, overcrowding, relaxed access to antibiotics, lack of resistance surveillance systems, and poor sanitation practices are some of the more important drivers of antibiotic resistance [43]. Recent studies have also shown that untreated hospital wastewater can contain high doses of antibiotics, which can drive selection for antibiotic-resistant genes in potential pathogens [44,45]. Therefore, it is recommended for future research to identify key challenges in combating antibiotic resistance in high-risk regions and to investigate which strategies would be the most effective and efficient to implement.

Our study also showed that colistin, often with a combination of synergistic drugs, emerged as a viable therapeutic strategy for PDR-PA; colistin was most often administered via the intravenous route [21,32,37,40]. However, as there currently is a dearth of literature on PDR-PA management and outcomes, it is recommended to gather more data through clinical trials to find the most appropriate antibiotic cocktail and dose. Aside from this, it is also advisable to develop novel medicines to maximize our options in combating antibiotic resistance.

Another concerning finding was the evidence of coinfections with PDR-PA, notably Acinetobacter and Klebsiella species. Polymicrobial infections can decrease antimicrobial effectiveness, thus making disease control increasingly difficult for clinicians [46]. Recent microbial and molecular studies might explain why these pathogens often coexisted and are co-isolated: certain strains of *A. baumannii* and *P. aeruginosa* are more resistant to a greater number of antibiotics and can secrete enzymes that confer antibiotic resistance. In exchange for protection against antibiotics and microbial competition, *K. pneumoniae* can provide the essential metabolites for the growth and proliferation of *A. baumannii* and *P. aeruginosa*, thereby increasing their collective survival and virulence [47-49]. This obligate mutualism phenomenon is called “cross-feeding and cross-protection” [47].

Therefore, with the substantial likelihood of PDR-PA having coinfecting microbes, it might be more prudent to test and provide coverage for other infections. This is especially true for *K. pneumoniae*, *E. coli*, and *A. baumannii*, which are the most common pathogens in intensive care units and have historically caused high mortality rates [50,51]. Dosing strategies to cover PDR-PA and co-pathogens while minimizing side effects must also be investigated in future research. 

Our review reveals that data on clinical outcomes of PDR-PA, particularly mortality and prolonged hospitalization rates, is still limited. Given the relative rarity of PDR-PA, five studies had particularly low sample sizes (n < 30), and important risk factors like prior infectious episodes and previous antibiotic or steroid use were not adequately described. Furthermore, we are unable to eliminate the risk of bias stemming from the variability in methods of susceptibility testing: two studies used the Vitek 2 system instead of the more popular disc diffusion method, and only three studies utilized broth microdilution (Table 1), which is the recommended method for testing polymyxin resistance [52].

Despite these limitations, this is a comprehensive review of the risk factors and clinical characteristics of patients with PDR-PA. To our knowledge, this review is the first to demonstrate the extent to which PDR-PA can be found in acute and severely ill patients. Moreover, our findings suggest that those at high risk for colonization of PDR-PA could benefit from empiric treatment that would also cover the more common co-infecting pathogens. 

## Conclusions

Over the past number of years, *P. aeruginosa* has become a notorious nosocomial pathogen not only for its increased prevalence in acute infections and severely ill patients but also for its extensive mechanisms for antibiotic resistance. This paper describes the increasingly concerning PDR-PA, its frequency in burn, CF, and immunocompromised patients, its propensity for co-infections with other gram-negative bacteria, and the preference for the antibiotic colistin as a therapeutic regimen. To prevent the spread of PDR-PA, increasing surveillance and improving environmental decontamination must be done, along with pursuing further research on the development of novel therapeutic strategies.
